# “Caring for insiderness”: Phenomenologically informed insights that can guide practice

**DOI:** 10.3402/qhw.v9.21421

**Published:** 2014-01-21

**Authors:** Les Todres, Kathleen T. Galvin, Karin Dahlberg

**Affiliations:** 1 School of Health and Social Care, Bournemouth University, Dorset, United Kingdom; 2 Faculty of Health and Social Care, University of Hull, Yorkshire, United Kingdom; 3 Sahlgrenska Academy, Göteborg University, Sweden

**Keywords:** Caring, person centred, phenomenology, lifeworld, humanization, individualized care, reflective analysis

## Abstract

Understanding the “insider” perspective has been a pivotal strength of qualitative research. Further than this, within the more applied fields in which the human activity of “caring” takes place, such understanding of “what it is like” for people from within their lifeworlds has also been acknowledged as the foundational starting point in order for “care” to be caring. But we believe that more attention needs to be paid to this foundational generic phenomenon: what it means to understand the “insiderness” of another, but more importantly, how to act on this in caring ways. We call this human phenomenon “caring for insiderness.” Drawing on existing phenomenological studies of marginal caring situations at the limits of caring capability, and through a process of phenomenologically oriented reflection, we interrogated some existential themes implicit in these publications that could lead to deeper insights for both theoretical and applied purposes. The paper provides direction for practices of caring by highlighting some dangers as well as some remedies along this path.

Tolstoy and Chekov were on a walk in the spring woods when they encountered a horse. Tolstoy began to describe how the horse would experience the clouds, trees, smell of wet earth, flowers, sun. Chekov exclaimed that Tolstoy must have been a horse in a previous life to know in such detail what the horse would feel. Tolstoy laughed and said: ‘No, but the day I came across my own inside, I came across everybody's inside’. (Sutherland, 2013, p. 30)

Understanding the “insider” perspective has been a pivotal concern for any applied field where the human activity of “caring” is important. Such understanding of “what it is like” for people from within their lifeworld has been particularly acknowledged as a foundational starting point for health-related caring (Toombs, 2002). Indeed, the notion of appreciating or understanding a person's “insiderness” has been cited as one of eight essential dimensions of what it takes to “humanise care” (Todres, Galvin & Holloway, 2009, p. 3): “To be human is to live in a personal world that carries a sense of how things are for the person.” However, we believe that more specific attention needs to be paid to this foundational phenomenon in relation to caring: what it means to understand the “insiderness” of another, and more importantly, how to act on this in caring ways. We call this phenomenon “caring for insiderness.” There are many qualitative studies already in the public arena that, although not directly focusing on the phenomenon we name, could be harnessed and reflected upon in order to deepen our understanding of this concern. So we posed the question: In what ways can the findings of research help us in arriving at further useful insights that have implications for practice? In this paper, we turn to seven published phenomenological studies with the following reflective question in mind: What does each study tell us about the phenomenon of “caring for insiderness”?. We wanted to pay attention to some rich sources of phenomenological research that already exist. This is not a systematic literature review but rather an analysis of a group of papers that served to reveal some new depth and detail about “caring for insiderness.” We believe that there is not enough reflection on the meaning and implication of what sometimes looks like unrelated studies, but which can be meaningfully related to one another when one takes a fresh perspective and where one has a particular human phenomenon in mind. Within this spirit, we chose seven articles that used a phenomenological approach to research and which focused on a range of issues about the challenges and dilemmas of caring in a variety of settings. We chose phenomenological research because we have found that such studies usually express their findings in ways that often connect to existential human themes that far transcend the specific focus of the paper and are thus amenable to interrogation with a different focus in mind. Such “amenability” of phenomenological research to related existential interrogation makes sense to us because of the epistemological underpinning of phenomenological research that acknowledges the “seamlessness” and “interconnectedness” of lifeworld phenomena. Further to this, in our choice of papers, we were particularly attracted to marginal caring situations at the limits of caring abilities. These extreme situations called our attention to the outer boundaries of what needs to be included within the phenomenon: “caring for insiderness,” and as such, posed interesting challenges for any situation in which an appreciation of another person's “insiderness” is approached. Regarding our style of reflecting on the study, we engaged in such a way as to become present to implicit meanings being revealed which we put into language. We now report on a number of insights that emerged through the range of the chosen research studies. The studies are summarized in Table I.

**Table I:** Summary of phenomenological studies

Author and date	Focus of study and research question	Mode of access to the phenomenon and from the perspective of:	Methodological approach	Sample	Range of phenomena relevant to “Insiderness”
Stenwall E, Sandberg S, Eriksdotter Jonhagen M, & Fagerberg I (2007)	Encountering the older confused patient: professional carers’ experiences. The aim was to gain an understanding of the meaning of professional carers experiences of their encounters with older confused patients	Professional carers were interviewed using a phenomenological approach	Descriptive phenomenology	Ten professional carers from wards specializing in caring for confused older patients in Sweden.	• Concentrates on insiderness that is opaque, unforeseeable and unpredictable • Understanding another's insiderness by exchanging self with other
Lundqvist A, Nilstun T, Dykes AK (2002)	Mothers’ experiences of professional care when their newborn dies	Mothers were interviewed 2 years after the death of their newborns	Hermeneutic phenomenology	Sixteen mothers who had lost their baby within 2 weeks of birth Sample drawn from three Swedish hospitals	• Showing understanding of others insiderness reduces isolation and increases patients power • Conversely, iatrogenic suffering that comes from a lack of showing this • The importance of a non-judgemental reaching out without having to be asked
Stenwall E, Jonhagen ME, Sandberg, J Fagerberg I (2008)	The older persons experience of encountering professional carers and close relatives during an acute confusional state	Older people who suffered acute confusion were interviewed following an initial introductory meeting	Phenomenological oriented latent qualitative analysis of interview data	Seven older patients all over 65 from two geriatric wards in an emergency hospital in a metropolitan area	• The distance and loneliness of ‘being far away’ from one's insiderness being understood • Reaching out to insiderness as a general move—the feeling “of being taken seriously”
Carlsson G, Dahlberg, K, Lutzen K, Nystrom M (2004)	Violent encounters in psychiatric care	To deepen understandings experiences of violent encounters	Reflective lifeworld approach using “re-enactment interviewing” about an event chosen by participants and written narratives about a positive or a violent encounter from each participant	12 staff from psychiatric and community care in Sweden: one nurse and six care assistants from psychiatric care and one nurse and four assistants from community care.	• Insiderness as a foundation for authentic care • Experiencing the abandonment of insiderness when coming from a “blank face” • Acceptance of the another's vulnerability is an important dimension of caring for insiderness
Nystrom M (2006)	Aphasia—loss of the world of symbols and existential loneliness	The experience of aphasia and professional care giving. Interviews with patients with speech difficulties	Reflective lifeworld Hermeneutic analysis of interview data.	Four women and five men 45–72 years old.	• Insiderness is worth little if it can't come outside • How insiderness always needs to be in play with a participation in outsiderness or otherness
Nordgren et al. (2008)	The meaning of caring support from perspective of women in middle age living with heart failure	The meaning of caring support. Interviews with women living with heart failure	Descriptive phenomenology	Six women	• An appreciation of insiderness helps to wisely shift care from an impersonal anonymous level to an existential personal level
Norlyk & Harder (2009)	The lived experience of fast track surgery. Patients’ experiences of early discharge following bowel surgery	The experience of recovery following early discharge. Interviews with patients in their own homes 2 weeks after early discharge. Patients asked to describe what it was like to live through the recovery period	Descriptive phenomenology	16 patients 53–77 years. All interviewees who had been discharged within 5 days following bowel surgery	• A focus on what happens when external protocols are privileged over caring for insiderness • The dilemma of how to come to terms with a situation when an outside perspective of themselves needs to be privileged

## Empirically informed insights into the phenomenon: “caring for insiderness”

a) An extreme example of the receding depths of insiderness in a caring context: a meaning revealed by professionals who cannot see a confused patients’ insiderness.

Stenwall, Sandberg, Eriksdotter Jönhagen, and Fagerberg (2007) explored the professionals’ experience of encountering older confused patients. Their study points to a particular nuance of insiderness, which concerns the dilemma that a professional may experience when encountering a confused person. Here, the professional is challenged by having to open themselves to an experience of unfamiliarity. The challenge that this poses is how to reach and understand the confused patient's “insiderness” when this “insiderness” is so opaque or immediately unfamiliar to the professional. The confused person's actions are unforeseeable and their words unpredictable. The authors of the study provide good descriptions of the professional carers’ understandable feelings of insecurity when there is such an extreme lack of access to a coherent sense of the patients’ experience of life from the inside.

The way that the professionals proceed is to attempt to “use themselves” by exchanging “self” with “other,” through a process of trying to imagine what they would have said or done in a similar situation, and what they would have meant with similar words and actions. This experience of reaching towards insiderness is “an attempt to make contact to something within the patient that the professional carer can trust” (p. 518). In other words, it is a search for some degree of “closeness” in the sense of trying to imaginatively contact the common humanity between carer and cared for: “they seek something within the patient to get in contact with” (p. 520) and “they seek confirmation from the patient” (p. 519). At the same time, there is always a certain distance for professional carers, in that they can never be sure that they have achieved a coherent understanding of the confused other's insiderness. This experience therefore opens up a certain kind of vulnerability in the professional carer which is unavoidable because, in this situation, they have little “knowing” to rely on, especially when they “fail to get close to the patient.” The study reveals different possible pathways in response to this dilemma of “not knowing.” The first possibility is that the professional, in being excessively focused on worrying about the safety of the person, attempts to gain control in the encounter and intervene on the others behalf in order to guide the patient towards a predetermined purpose. This predetermined purpose however cannot be said to be the patient's purpose, but rather the professional's purpose. Such a pathway abandons a meaningful consideration of the insiderness of the patient because of safety concerns. The professional then gains a certain sense of personal security and a relief of their own anxiety, but this is at the cost of losing the confused person's purpose and of letting go of the attempt to reach towards the other's insiderness in such difficult circumstances.

Another nuance in response to not being able to easily find the patient's “insiderness” is what Stenwall et al. (2007) call “always being on guard.” In “always being on guard”, the carers “feel responsible for the well-being of the patient and have to guard the patient against themselves” (p. 518). However, this feeling of responsibility is an ambiguous experience for the professional in that it is not without conflict. Such professionals report feeling guilt and fear about taking so much responsibility in such an interventionist way, especially when the patient is no longer in a confused state. They worry in such situations that they have failed to care for the patient's insiderness.

The extreme nature of the example of marginality reported in this study is that professional carers can never fully feel sure or secure about successfully achieving a sense of insiderness of the other. Thus, personal “insiderness” always recedes from another's view. So, the issue for the professional carer in this vulnerable situation of not knowing is that they have to continually manage “the not knowing of insiderness” and its on-going uncertainty. All they have got is themselves trying to reach some sense of the insiderness of the other, and this trying to reach “that” requires them to feel a minimal but necessary degree of feeling safe and secure, because if they don't feel a minimal degree of safety and security, then the alternative path is an excessive need to take control.

This first theme announces the dilemma and ambiguity of a sense of insiderness. The next theme unfolds in more detail this positive need for one's insiderness to be understood from the point of view of the “cared for person” in all its possibilities and range.

b) An example of how one's insiderness appears to seek or demand recognition and affirmation in caring situations: meanings revealed through the experience of older people being confused as well as through mothers experiences of their babies lives being threatened.

In a further study, Stenwall, Eriksdotter Jönhagen, Sandberg, and Fagerberg (2008) explored the older person's experience of what it was like encountering professionals and close relatives while they were going through or in an acute confused state. This study highlights what it is like from an older person's point of view when trying to make themselves feel understood and are unable to do so. When “far away” from the capacity to show their insiderness such older people can feel like “an outsider” and concomitantly experience loneliness. They feel imprisoned within a negative cycle of being misunderstood and cannot “find their own way out.” In such situations, the confused older person depends on the other to make sense of the situation. The study provides examples of how the confused person comes to feel safe and supported. From their side, the older people are acutely appreciative of “being reached towards” in the way discussed in the previous section. This general feeling of being “reached towards” makes older people feel that their world is being taken seriously; this is a great consolation, confirming that their insiderness matters even when it is not fully understood in detail.

A further example of how one's insiderness appears to seek or even demand recognition is provided by Lundqvist, Nilstun, and Dykes (2002). They examine and illuminate mothers’ experiences and perceptions of the care provided by neonatal staff (nurses, midwives and physicians), where a baby had died or where a baby's survival was threatened. The explicit focus by the authors of this study concerns how mothers felt both empowered and powerless at different times. Their findings draw attention to the things neonatal staff do which make mothers feel empowered and conversely, the things that make mothers feel disempowered, discouraged and even violated.

In relation to illuminating the phenomenon of insiderness, this study graphically illustrates how a professionals’ appreciation of a mother's insiderness can be very important in helping the mother feel understood, thus significantly reducing mothers’ sense of isolation and disconnection. Conversely a lack of appreciation of mothers’ insiderness can profoundly disempower them, leading to great iatrogenic suffering. The value of this study for illuminating the phenomenon of insiderness is in how it shows the pivotal role that power plays in facilitating or obstructing the pathway towards a care for insiderness.

When the mothers experienced a sense of recognition of their struggle from staff, mothers felt much closer to them and this closeness comforted the mothers. This experience made the mothers feel more able to make decisions about their baby, which in turn enhanced and supported mothers’ confidence and a sense of being more in control of the situation. Again, as in previous examples, caring for insiderness does not depend on a detailed understanding of the persons’ situation or world but rather on a non-judgemental, general “reaching out” to them, without having to be asked. Here, the mothers’ experience of a sense of benign closeness supported confidence. Within this marginal context, caring for insiderness is caring for what the mother is going through. This study also illuminates an interesting variation by which mothers responded to their insiderness not being understood or recognized by a kind of rebellious “taking of things into their own hands.” This is a kind of strength that these mothers achieved through facing not only the experience of their suffering, but by doing this in the context of feeling unrecognized or ignored. In such moments mothers (or patients in general) may “go with what they want” and find other allies for example, a partner or husband who is able to support them. The extreme marginal situation that this study focuses on, reveals the creative potential that can occur when one's insiderness is not recognized or understood, strengthening the persons’ autonomy, as well as the devastating potential of one's insiderness not being recognized, leading to experiences of violation. So, such recognition or non-recognition has a depth of power that can easily be missed. If a professional is not aware of the depth of the power they carry in such marginal situations, such power can be highly destructive.

c) An example of insiderness calling for understanding of unique existential concerns and meanings: a meaning revealed through women's expressed needs of living with heart failure.

Nordgren, Asp, and Fagerberg (2008) aimed to understand the meaning of support for women living with heart failure. The findings share some of the essential meanings of the previous study, that is, that formal care can become excessively caught up in routines, and that this can result in patients feeling angry, sorrowful or despairing about the failure of the care regime meeting them as unique individuals with their own variable concerns and needs. In relation to the phenomenon of caring for insiderness, this study highlights how in an on-going life-threatening condition, an adequate understanding of insiderness would focus on the experience of *existential uncertainty* rather than just the details, for example, symptoms, of the person's illness. This study portrays how women living with heart failure in middle age emphasized a wish to be responded to at a more existential level regarding the challenges of their illness and how this fitted into what it meant for their major or minor life projects (“being able to”). At best, they were frustrated that support and information was only directed to them in very general ways. They felt abandoned because the illness affected their whole life, but the professional focus was only on part of their life. The women wanted a more existential concern to be addressed, in which uncertainty was at the heart of the matter. They wanted to discuss things like how to find balance in their lives, in a context where their heart failure limits life projects. They wanted a discussion about daily life.

This study tells us about how an appreciation of insiderness opens up the core issues to be responded to (in this case, living a life of uncertainty) rather than mere external “facts,” and why existential issues need to be a crucial reference point if we want care to truly meet a human being.

d) An example of insiderness as a unique insistence of the body: a meaning revealed by professionals over-focus on external and standardized protocols.

Norlyk and Harder (2009) describe the lived experience of early discharge from hospital after colon surgery. Patients had participated in a fast track programme where they were discharged from hospital between 2 and 5 days after surgery. The authors were specifically interested in illuminating what it was like to live through the recovery period in the early days. In relation to the phenomenon of caring for insiderness, this study is revealing in that it highlights a marginal situation in which external protocols are privileged over caring for insiderness because of the perceived justification that it is what the marginal situation requires.

In this example, as in other marginal situations, such as intensive care, where the person “hands over” their entire bodily care to another, the body's recovery is seen as a homogeneous process. That is, institutional expectations are based on an enactment of recovery protocols that would work better if everyone was the same. Within such a regime “care” contains an emphasis on rules that patients need to follow (must do's). This tacit preference for everyone “being the same” highlights the absence of a particular meaning of “insiderness,” that is, the uniqueness and difference of each person's body and world. The study documents how patients lived a certain tension between the rules that are part of the postoperative care system and their own “insider experience” of what they felt they needed to recover. Post-operative discomfort created a dilemma for patients: of following the professional's advice regarding regimen objectives or responding to their own intuition of what could contribute to their comfort in a unique way. For example, unpleasant symptoms made patients feel weak, yet hospital norms and expectations often required them to be stronger than they could be when they were recovering. In the background, there is a strong desire to try and comply with professional expectations, that they be strong and co-operative. Part of the dilemma is there are times when an external expectation is an important part of the patient's recovery, and here they are required to have some faith in the professional's experience. Here they may be asked to exert some effort in ways that may go against their own inner sense of what is comfortable. The authors express one of the meanings of this as follows: “It took courage from the patients to ignore their body's signs and have complete trust in the professional assurances that their body would not be overworked by following the regime” (p. 176). But, on the contrary, this external demand was often overdone.

In relation to meaningful care, this study raises a very important question about the virtue of empowering “insiderness.” It illustrates how in some marginal situations where the insider perspective cannot be privileged, even then, the experience of care would be improved by always including a perspective which balances the external protocol with an appreciation of unique insiderness. Patients want staff to acknowledge their discomfort, to see that they are trying hard and want staff to believe in them. In this study, patients felt that they could not trust professionals if they as patients were not involved in a minimum of vital decisions; it is a balance.

e) An example of insiderness as an insistence to participate in the world: a meaning revealed by aphasic patients not being able to express themselves in everyday language.

Nystrom (2006) aimed to analyse the existential consequences of aphasia and what it was like for patients to struggle to regain the ability to communicate. This paper has many important and interesting themes, but we focus here on those dimensions of the paper that add insights that are relevant to the meaning of caring for insiderness. People experiencing aphasia become really worried about being considered stupid by others. The study graphically illustrates how, for these people, being aphasic is not only a humiliating experience but also condemns them to a deep existential loneliness and even a sense of imprisonment. Although this group of people share a sense of abandonment with people from the particular studies discussed previously that focus on the experience of confused older people, there is an important distinction. It is clear from Nystrom's study that the people living with aphasia have great clarity about their insider world, which they would like to share and which they cannot. There is a “shock” to this kind and level of existential loneliness, like being a ghost who is there but who cannot participate. In relation to the meaning of caring for insiderness, this study highlights how one's sense of one's own insiderness can be very coherent but is worth little if “it” can't come outside. The patients’ sense of existential loneliness constitutes a painful sensitivity that the health care system has abandoned them. This sense of existential loneliness is also heightened by their perception that other people misunderstand their aphasia as an incapacity to think. Such perceived judgements from others add a sense of a “loss of dignity” to their existential aloneness.

The poignant dilemma experienced by this group can teach us something about how insiderness is an important human essence, but can never move alone, and always seeks to be in play with a participation in “outsiderness” or otherness. Although some of the previously discussed studies may emphasize the importance of respecting the privacy and dignity of insiderness in some contexts this particular study indicates another “energy” of insiderness which is “to want to come out” and be understood and therefore participate in-the-world-with-others.

f) An extreme example of how the insistence of insiderness can even lead to violence if a sense of felt isolation is neglected: a meaning revealed in the psychiatric care setting where patients become violent.

Carlsson, Dahlberg, Ekebergh, and Dahlberg (2004) focused on situations in which patients became violent within a psychiatric care setting and the implications for professional caring. The authors of the study emphasize the tension between authentic personal care and detached care.

This study learnt from patients some important things about the kind of encounter that would help them to avoid the desperation of violence. Patients experiencing such care depended on professionals showing themselves in human ways, actively engaged and affected and moved or touched by the suffering of the patient. Here the patient also experienced a respectfulness of their insiderness which included a recognition and acceptance of the patient's vulnerabilities and shortcomings. The appreciation of insiderness has within it a concern for the well-being of the patient and this constitutes a caring power. Conversely when the patients experienced a certain detached instrumentality from professionals this would result in feelings of insecurity, fragility, vulnerability and abandonment. These feelings would feed into an already existing fear and sense of violation. A patient expressed what it was like to be met in a cold and distant way: “my lasting memory from these moments is the encounter with an expressionless blank face, with expressionless cold eyes staring back at me” (p. 295). This is “the face” in which the insiderness of the other has been abandoned. In the presence of such an encounter, patients reported feeling “reduced” to being a patient and further to feeling degraded and unworthy, helpless, disrespected, incapacitated and unimportant. The authors provide examples of how such extreme feelings of desperation can lead to episodes of violent reaction. This study therefore bears testimony to how abandonment of insiderness can become a matter of felt psychological survival.

## Implications for practice

In this section, we elaborate on the following three insights that emerged from our consideration:

- The importance of the understanding that “insiderness” always recedes from the view of the other so that it can never be grasped absolutely.
- How “reaching towards” the “insiderness” of another as a process and practice is often more important than “knowing” the details of someone's “insiderness.”
- That “caring for insiderness” calls for the complex use of self through “reflective openheartedness” and through lifeworld knowledge as an interpretive touchstone.

## “Insiderness” recedes from the view of the other

One of the central insights about the phenomenon of caring for insiderness that has been revealed by the analysis has to do with the challenge of the unforeseeable encounter. The contributions of Levinas (1969) provide an ontological context for the depth of such an unforeseeable encounter. In his work, he unfolds what he refers to as “the infinity of otherness.” One of the implications of this is that there is an epistemological limit to what we can know about any other person, and indeed if we were to prematurely lurch towards “such knowing” we will have breached an ethical calling that keeps the “infinity” of the other alive. Levinas refers to “the face” as a metaphorical signification of the world of the other that can never be summarized or reduced to something we assume to know: “The face resists my possession, resists my powers” (Levinas, 1969, p. 197). And further: “I can recognize the gaze of the stranger, the widow, and the orphan only in giving and refusing; I am free to give or to refuse …” (Levinas, 1969, p. 77). The confused patient presents a particular example of the infinity of otherness. We have seen how the professionals, if they are unable to work with this existential limit, can close down by either rushing for ways to control the other or alternatively towards a premature abandonment of the other. Striving towards insiderness within the context of the infinity of otherness can only occur by the use of oneself rather than the application of already made knowledge. In other words it is only by using oneself that one can make reflective decisions in the moment. For the professionals such a “use of oneself” in the moment is full of vulnerability because they are so uncertain about the insiderness of the patient. This “existential given” (that insiderness always recedes from others so that it can never be fully grasped absolutely), poses a fundamental question about the nature of what evidence could mean in lifeworld-led care.

## “Reaching towards” otherness as more important than “knowing”

The status of “evidence” is humbled by the unique situations of practice. In our conventional professional discourse on evidence-based practice, we believe that an unrealistic fantasy has been set up that privileges the discourse on evidence over a discourse on understanding and judgement. *The* *process* of “reaching towards” insiderness may be more important than an accurate understanding of what is *in* the insiderness (that is, the content of the knowledge that is found there). Thus, a *reaching* for insiderness is more of a productive direction in practice rather than a fully achieved outcome.

Consistent with this insight, the findings of our review motivates us to suggest that the achievement of understanding “insiderness” is always vulnerable rather than something that can be achieved as a completed outcome: that “reaching towards” as a process is more important that the achievement of “knowing” as an outcome. By its very nature, caring for insiderness is always more intricate and more specific than any generality, as it sits within the uniqueness of the receding nature of insiderness (Gendlin, 1991; Polkinghorne, 2004). Although “general understandings” may *sensitize* us for reaching towards insiderness, these general understandings need to be bridled by the openness of practice and by the many nuances that all encounters with patients contributes. Such openness of practice is by essence “always on the way” and is best defined by the “energy” of “reaching towards” rather than an attitude which forecloses the complexity of its task.

## The alternative: caring for insiderness through the complex use of self

If this study has revealed insights which expose the vulnerability of sense-making in the unique encounter, then the question arises: how can professionals authentically care for insiderness given the existential limits we have been articulating. We propose two ways: a) reflective openheartedness and b) lifeworld knowledge as an interpretive touchstone.

a) Reflective openheartedness

The analysis in this paper suggests a certain emotional and embodied capacity that is important when caring for the insiderness of another. There are occasions where carers need to feel a minimal but necessary degree of safety and security about what is going on in relation to the patient or another cared-for person. This raises the question of how carers are able to emotionally tolerate a degree of “not knowing” in order to sensitively reach towards insiderness in openhearted ways without too much preconception.

How can this “openness to not knowing,” however, not be enacted as a form of abandonment? The stories of the mothers where their insiderness was not understood or recognized resulted either in a rebellious “taking of things into their own hands” or a sense of traumatized violation from which they found it difficult to recover (Lundqvist et al., 2002). So, betrayal of insiderness can either force independence in a productive way or else can traumatize in a destructive way. Carers who are attuned in an open-hearted way manage the emotional tension within themselves between encouraging, or even challenging, independence and that of a nurturing acceptance which is non-demanding. The particular emotional quality that underpins “reaching for insiderness” is one of patience and anticipation.

Meaningful caring for insiderness thus challenges a carer to stretch towards “the wisdom of insecurity” (Watts, 2011). Reaching towards insiderness involves the use of the self by carers in such a way as to “hang out” at the edge of their own levels of security. One needs to find this edge where one neither completely gives up one's desires for security, drawing on sources of knowing, but on the other hand stretches towards an open-minded questioning in the freshness of the moment. This emotional capacity (Galvin & Todres, 2009) is particularly germane to attending to the task of the receding nature of insiderness and for the *care* of this receding nature.

b) Lifeworld knowledge as an interpretive touchstone

The analysis in this paper suggests that there are a number of specific understandings that are important when caring for the insiderness of another.

- If a carer or professional is unable to specifically understand the details of another's world then it is important to try to imaginatively understand something more general as a focus, namely, the common humanity between them. This requires a shift in cognitive focus from the “other” in him or herself to an understanding of what is between them at this moment. So for example, instead of imagining what the other person is thinking, to rather shift focus to an awareness of something that the carer and the other person may be sharing at that moment, and to articulate that.
- Patients’ need for recognition and the strong striving towards seeking recognition suggests that we can underestimate how much the cared for person is looking for someone *to trust* in a general sense and that this often does not require the carer to know all the details. Carers need to understand that when they say “I understand” this does not have to mean that they specifically understand the content of the person's “insiderness,” but it rather has the form: “I understand your vulnerability at the moment.” Here carers understand the great power that they hold in being the one who engenders trust rather than being the one “who knows.” In a lifeworld-led approach this is a shift of understanding away from a personal expectation of certainty towards “the reaching” towards the “ambiguous” human situation.
- Our analysis further suggests a consideration of the *existential meaning* of what the cared for is going through in general. Such understanding is shown by a response which acts on the *implications* of what the person is going through. In other words, it involves a focus on what the person is able to do or can't do in their everyday lives and to help the person to do something that anticipates their needs. Boss (1963) calls this “anticipatory care” as opposed to “intervening care.” In other words, even though carers may not be able to understand the persons inside world in detail, they are able to become imaginatively sensitive to what the person is trying to “reach for.”
- Another kind of general understanding that may benefit carers when they can't have specific understandings of a person's insiderness refers to an understanding of the kind of *bodily struggle* that a person is going through in their illness trajectory. This focus can be particularly empowered by *sensitive observation* of these bodily struggles and what this might mean for the unique ways that a person's body is signifying about their situation. This is a shift of focus from the mere bodily behaviour or symptoms to an understanding of what lived body is trying to say about the person's insiderness. An overreliance on the body–object gaze may shift attention away from insiderness. For example, when the person is expressing discomfort, the carer focuses on the insiderness-generated message about what this is *uniquely* saying in that moment rather than how it fits into a view of this from a diagnostic or categorized framework. Such a focus enables the carer to reach out by putting into words that which is implicitly meaningful in the person's body and behaviour. In this way, the carer will help the person to listen to her/his body. Part of this is that the carer will embrace a person's fears about her/his bodily symptoms using a language that does not over-categorize or objectify. By staying close to embodied experience of the other, meaningful reassurance and a way for the patient to “move forward” can be offered.
- Finally, we argue the need for shifting one's focus of understanding from the cared-for-in-themselves to a focus on the cared-for-in-relation to others. The focus of understanding thus becomes relational in its gaze and acknowledges how the cared-fors’ condition is partially constituted by how they are treated by others. In this view “illness” is not separate from how a person's insiderness is understood or ignored. In two of the analysed studies we saw how deep or central the “welcoming” of insiderness was, or how deep and central the “abandoning” was (Carlsson et al., 2004; Nystrom, 2006). The course of the illness in both contexts is not independent from how it is responded to by others. This kind of lifeworld understanding thus requires a participative world view in which brute facts such as illness are always seen within relational contexts that can change them. This understanding thus emphasizes the great power of “reaching for insiderness” as a force for healing, and how its absence can become a form of iatrogenic suffering.

In conclusion, it could be said that the “insiderness” dimension of our humanity is the “soft underbelly” that often lies hidden in the shadows. It is both the place that hides our vulnerabilities and therefore often the place that is neglected in our discourses. The alternative to such neglect would be a much keener attunement to the phenomenon of “caring for insiderness” as a foundational requirement for care *to be* care. It is hoped that by restoring “insiderness” explicitly to its essential status for meaningful caring, something has been contributed about what it is, what “reaching” for it means, and what the consequences may be if it is sufficiently neglected.
